# ‘We can go hand in hand’ – patients with COPD and their experiences of relational continuity of care in Swedish primary care: a qualitative study

**DOI:** 10.1080/02813432.2025.2608119

**Published:** 2026-01-20

**Authors:** Sara Roos, Malin Sjöström, Jörgen Medin, Christina Melin-Johansson

**Affiliations:** a Faculty of Health Sciences, Mid Sweden University, Östersund, Sweden; b Lugnviks Health Care Center, Region Jämtland Härjedalen, Östersund, Sweden; c Department of Public Health and Clinical Medicine, Umeå University, Umeå, Sweden

**Keywords:** Continuity of care, COPD, experiences, patients, primary health care, stigma, trust

## Abstract

**Aim:**

This study aimed to explore how patients with chronic obstructive pulmonary disease (COPD) experience continuity of care through their encounters with a specialised asthma/COPD Registered Nurse in primary care.

**Design:**

An inductive qualitative study using interpretive description.

**Methods:**

Data were collected through semi-structured individual interviews with 13 purposively sampled patients with COPD from four regions in Sweden. The audiotaped interviews were conducted over a 5-month period (September 2024–January 2025) *via* telephone, transcribed verbatim and analysed using interpretive description.

**Results:**

Three themes describe the results. *Meaningful care when encountering a dedicated nurse*, which patients describe when encountering the same trusted nurse. *Being acknowledged and listened to* highlights the patient’s appreciation for the nurse’s knowledge and competence, both regarding the patient and the disease. *Feeling trust in the relationship*, a personal but not overly private relationship that fosters trust, which is essential since COPD can be a shameful disease. Some patients haven’t shared their diagnosis with loved ones, making the nurse a vital and comforting point of contact.

**Conclusion:**

Patients with COPD value continuity of care with a specialised asthma/COPD Registered Nurse. Trust and a non-judgmental relationship are essential for continuity to be experienced positively. Continuity is more than repeated encounters; it requires quality interactions that empower patients and address the stigma associated with COPD.

**Reporting Method:**

Consolidated Criteria for Reporting Qualitative Research guidelines.

## Introduction

Continuity of care (COC) is defined as the experience of patients receiving consistent and connected care over time [1]. Three different types of continuity are defined: *Relational continuity* is based on an ongoing relationship with a trusted healthcare professional, which provides the patient with a sense of predictability and coherence. *Informational continuity* is described as knowledge about the patient. *Management continuity* is defined as the flexibility to adapt to changes in patient care [1]. In primary care, relational continuity is particularly valued, as it is associated with higher patient satisfaction and improved care quality [1–4].

Chronic obstructive pulmonary disease (COPD) is one of the leading causes of death worldwide, with an estimated global prevalence of 10.3% (Global Initiative for Chronic Obstructive Lung Disease [5]. There is a European agreement on treatment goals and diagnostic criteria for COPD, although differences remain regarding patient subgroups and treatment recommendations [6]. In Sweden, the majority of patients with COPD are diagnosed, treated, and followed up in primary care, where specialised asthma/COPD Registered Nurses (RNs) often provide patient education and annual check-ups in accordance with national guidelines [7].

Patients with COPD face complex care needs and might experience exacerbations [5], often compounded by feelings of shame and self-blame linked to their condition [8]. Both physical and psychological well-being may be compromised, potentially influencing family dynamics and the patient’s social interactions. COPD may also lead to a perceived loss of independence, accompanied by persistent anxiety over their deteriorating condition and breathlessness [9–11]. Hence, to ensure effective care in the nurse-patient relationship, the encounters should be structured around three fundamental components: a caring RN, a connection, and mutual trust. These three aspects define a caring encounter. Conversely, without trust, the interaction may feel impersonal and uncaring [12]. Patients with COPD need more information about their disease [13]. Fewer than half of the patients in a register study had received patient education [14]. However, patients tend to value the knowledge and support provided by a competent RN [15,16].

While much research on COC has focused on the GP-patient relationship [17–20], the role of RNs in ensuring continuity has been less explored [21,22]. However, studies in other areas have shown that when RNs listen to patients and take their views and agendas into account, patients perceive COC as valuable [23,24]. While some research has explored asthma/COPD nurses’ own perspectives on COC [25], to our knowledge, no previous study has specifically examined COC from the perspective of patients with COPD receiving care from specialised asthma/COPD nurse in primary care. Addressing this gap is important, as previous research shows that insufficient information, lack of trust, and limited nurse expertise can undermine patient care and disease management [26–28]. By clarifying the patient’s perspective, the present study contributes new knowledge that may inform both the understanding and organisation of care for individuals with COPD.

## Aim

This study aimed to explore how patients with COPD experience continuity of care in their encounters with a specialised asthma/COPD registered nurse in primary care.

## Method

## Design

This study was designed as an inductive, qualitative study with interpretive description (ID), as described by Thorne [29]. Semi-structured interviews were conducted with 13 patients with COPD who receive primary care. The Consolidated Criteria for Reporting Qualitative Research (COREQ) checklist was used for reporting this study [30].

## Setting

In Sweden, healthcare is organised into 21 self-governing regions that include both primary and secondary care [31]. This study was conducted in both rural and urban areas across four different regions in Sweden, from the north to the south. To gain insight into patients’ experiences with continuity of care, we aimed to include various primary care centres with an RN who specialises in asthma/COPD and participants with a wide range of experiences.

### Participants and procedure

A purposive sampling approach was used to recruit adult patients diagnosed with COPD who regularly receive care from an RN specialising in asthma/COPD management in primary care. Patients receiving specialist care in hospital units were not included. Written study information was sent to managers at eight primary care centres with established asthma/COPD nurse-led clinics. Four managers provided written consent for their centres to participate. Managers at the primary care centres received study information and consented to patient recruitment. A contact RN specialising in asthma/COPD at each clinic identified eligible patients and invited them to participate. Interested patients received written information and a prepaid return envelope for consent forms. Upon receiving signed consent with contact details, the first author (SR) called the patient to provide additional information, answer questions, and schedule interviews, ensuring respect for participants’ integrity and autonomy. Thirteen participants (5 males and 8 females) were included in the study. Each primary care centre contributed 2–4 patients. Thirteen informants were deemed sufficient, as adequate data had then been obtained [29,32]. The patient characteristics are shown in Table 1.

**Table 1.** Characteristics of study participants.

Characteristics	n
**Sex**	
Male	5
Female	8
**Age**	
61–70	4
71–80	7
81 or older	2
**Years with the diagnosis COPD**	
1–5	5
6–9	4
10–14	1
>15	3

### Data collection

All data were collected through individual semi-structured interviews conducted by the first author (SR) between September 2024 and January 2025. The interviews were conducted by telephone and recorded. The choice to conduct these interviews *via* telephone was for practical reasons due to long distances between the interviewer and the interviewee (as far as 870 kilometers). For this reason, all interviews were carried out by telephone, allowing for comparability. The interviews lasted between 24 and 59 min (mdn = 31 min). The interviews were transcribed verbatim by the first author after completion. A semi-structured interview guide (Table 2) created by the authors enabled the exploration of patients’ experiences of COC as provided by an asthma/COPD RN.

**Table 2.** Interview guide.

The initial question was presented in all interviews and structured as follows: Do you have a specific COPD nurse that you can turn to? What does continuity of care mean to you when you have a specialized COPD-nurse to turn to? What does COC mean to you? Is there something else there that is important? How do you experience encounters with the COPD nurse? Can you describe a good meeting and a less successful one? Is there something in the care for you that you would like to change?

### Data analysis

Data were analysed with Interpretive Description (ID) according to the principles described by Thorne [29]. ID was chosen for its exploratory nature, which makes it possible to explore patients′ experiences of COC. The analysis process started after the first interview was conducted by the first author (SR). A field notebook was also used by the first author (SR) during data collection and analysis to record thoughts, questions and ideas throughout the process. The field notebook was essential for capturing evolving insights and documenting the first author’s subjective experiences, thereby enriching the inductive research process.

The transcribed interviews were read and re-read by the first author to find descriptions that enabled new insights into COC. Relevant sections of texts were marked and described using a few words in the margin. After a few interviews were conducted, co-authors were involved in the analysis to determine whether any new insights needed to be clarified before continuing with the remaining interviews. However, no changes were made in the interview guide. Through an iterative process in the research group, broader themes emerged from patterns and relationships in the data. The text was divided into meaningful themes related to the patients′ experiences of COC. These themes were initially organised by the first author (SR) and subsequently summarised, reviewed, and refined through collaborative discussions within the research team. To highlight that the themes were grounded in data, the results were illustrated with quotes. An example of the coding process is shown in Table 3.

**Table 3.** The analyzing process in ID, examples.

Section of text	Code	Theme
It’s just like shopping for groceries. You shop in the same way and place. You think it’s good. That’s how you want healthcare to be too. Yes, it’s more convenient if you know it’s the same, otherwise you have to start over.	The same RN	Meaningful care when encountering a dedicated nurse
The nurse knows what I want to know, and I do not know if I need to know anything else. We can go hand in hand here, which I think I do with her. She knows what I need to know, since she listened to me and asked what I need.	Being listened to	Being acknowledged and listened to
It’s more about how it is perceived in society, as it is still shameful. I don’t know if that’s the only reason I didn’t tell my loved ones.	Shameful	Feeling trust in the relationship

## Ethical considerations

The study received ethical approval from the Swedish Ethical Review Authority (Dnr 2023-05034-01) to ensure that sample recruitment, data collection and handling followed good research practice. The World Medical Association′s Declaration of Helsinki [WMA] [33] guided this study, and participants′ confidentiality was prioritised. All participants gave written informed consent to be included in the study. The data were handled according to the General Data Protection Regulation (GDPR) [34]. The interview transcripts were stored in the author’s private catalogue and secured with personal passwords according to University policies to ensure good research practice.

## Rigour and reflexivity

Rigour was maintained by using Thornes′ [29] criteria of quality. *Epistemological integrity* was concluded through the co-authors’ guidance and feedback. *Representative credibility* was enhanced since the sample of participants was recruited from four different regions and primary care centres, and by the participants’ different levels of experience living with COPD and years since diagnosis. *Analytical logic* was reflected in the use of an audit trail to help with reflections and understanding of the dataset. *Interpretive authority*: Transcripts were prepared verbatim by the first author. Regular meetings were held among the authors to evaluate coding developments and foster the exchange of ideas. At these meetings, transcripts, notes and coding were shared. According to Thompson Burdine et al. [35], themes begin to develop as researchers immerse themselves in the data. The authors engaged in frequent discussions about emerging themes. Through this ongoing process of reflection and dialogue, patterns gradually emerged and were ultimately articulated and presented in the result below.

## Results

The analysis of interviews led to the development of three main themes. These themes reflected the experiences of patients with COPD in relation to COC in primary care with an RN specialised in asthma/COPD. In the results, the RNs specialised in asthma/COPD will be referred to as nurses (Table 4).

**Table 4.** Main themes.

Theme 1	Theme 2	Theme 3
Meaningful care when encountering a dedicated nurse	Being acknowledged and listened to	Feeling trust in the relationship

### Theme 1 – meaningful care when encountering a dedicated nurse

This theme refers to the fact that patients consistently expressed how valuable it was to have a specific nurse as their point of contact. Knowing who their nurse was and being able to picture them made the healthcare encounter more relaxed and secure. However, patients also emphasised that continuity alone was not enough; the quality of the relationship and the nurse’s engagement determined whether care felt meaningful.

Meeting a dedicated nurse who not only had knowledge of their medical history but also proactively followed up and coordinated their care was important.

‘And that I can contact her at any time if I run into problems. Having someone I can now picture in my mind is incredibly important. It′s so valuable to have a steady person I feel connected to, someone I can talk to about my situation’. P9

Even though information was available in the medical records, patients felt that meeting the same nurse repeatedly made the care encounter smoother and more personal. The patients did not have to repeat their story at every visit, which helped build trust and comfort.

‘It’s really important. Absolutely. Should I then sit down with someone new and start telling my whole story again? No, they (the nurses) have it on the computer, but it’s like they don’t even check it. No, it’s really important’. P10

The patients also expressed feeling welcomed, that there was no stress, and that the nurse genuinely cared about them. Patients also admired the nurse’s attentiveness, such as monitoring symptoms and evaluating medications. Patients described how their concerns shaped the agenda of the encounter, with nurses responding according to their individual needs.

‘But what I’m saying is that my new nurse takes care of everything; I’m impressed by their way of working. No, but it’s good, and she asks me about this disease. About the symptoms I have, if I feel anything special, how the medication is working, and she… yes, follow-up on most things, and I can’t ask for more’… P3

On the other hand, meeting a not-so-dedicated nurse was described as someone who simply went through a checklist, without any real depth or personal connection in the interaction. Some patients described previous experiences in which, despite having a designated nurse, the encounters felt shallow and impersonal. These follow-ups felt rushed, and the nurse did not seem interested or responsive to questions. As a result, continuity did not translate into meaningful care.

‘Yes, she probably knew, but she was a bit like that, I don’t know how to explain… but it was as if when I wanted to ask something, she looked so rushed at me, as if she had so many other things she needed to do after my visit’. P11

Patients compared earlier experiences to their current care, noting that earlier encounters with nurses were often superficial. Limited to routine checks like spirometry, without further engagement or explanation about managing their COPD. The patients realised only later that their care could be more comprehensive and supportive when they met a nurse who was involved in their care.

‘It’s just like shopping for groceries. You shop in the same way and place. You think it’s good. That’s how you want healthcare to be too. Yes, it’s more convenient if you know it’s the same, otherwise you have to start over’. P2

### Theme 2 – being acknowledged and listened to

This theme highlights the patient’s appreciation for the nurse’s knowledge and competence, both regarding the patient and the disease. The patients explained that the information provided by the nurse needed to be understandable and tailored, and that time was an important component. The patients expressed that the nurses possessed expertise in this area, and together with the patients, the right care could be achieved.

Being acknowledged by the nurse, which often stemmed from a sense of familiarity established over repeated encounters, was described as valuable. Patients felt that nurses had already developed a clear understanding of them through previous encounters, an understanding not observed among other healthcare professionals, who often lacked the time to build such relationships due to limited time spent together. This established relationship often enabled nurses to foresee the patients’ needs in terms of information and knowledge.

‘The nurse knows what I want to know, and I do not know if I need to know anything else. We can go hand in hand here, which I think I do with her’. P13

This familiarity reduced stress and made encounters more efficient, since patients did not need to carefully consider how to explain themselves each time. When care was based on general information rather than individual needs, important personal details were sometimes overlooked, something patients had experienced in the past.

‘I have got more personalised information now, I don’t have to explain what I have done the last five years or so since she already knows that’. P8

Patients appreciated being listened to and receiving guidance tailored to their specific needs and concerns, and that the information they received was personalised and relevant. This was particularly important in the management of their disease, when individualised advice—such as how to handle exacerbation or use inhalers correctly—was essential.

‘The nurse said for the first time that you shouldn’t take everything at once. You need to wait a while between the first inhalation and then wait a little longer. It would have made a big difference if you hadn’t received that information; it might not have had the same effect’. P2

At diagnosis, patients reported receiving no information about COPD. This improved when a nurse specialising in COPD joined the primary care centre. Patients valued the nurse’s expertise and appreciated the personalised support, which had previously been missing. They learned they could influence their condition through exercise and breathing techniques, information they hadn’t received before. Time spent with the nurse was key to receiving relevant guidance and feeling acknowledged.

‘It is better to talk to the nurse who takes care of everything instead of someone else, since she gave me all the information I needed. But without the time she gave me during the encounter, then I would have talked to someone else’. P13

Some patients carried emotional burdens from past experiences with family members who had COPD, which influenced their fears about the future.

‘COPD is a powerful word for me because my father had it. I’ve seen so many neighbours who have it and need to carry oxygen tanks with them in their wheelchairs. For me, it represents death. But when I got the information from the nurse, then I understood that I am not alone in this, I will get help’. P10

When patients were given the opportunity to talk about these issues, nurses who understood the context were better equipped to offer reassurance and explain that treatment options had evolved and that help was available for them.

### Theme 3 – feeling trust in the relationship

In this third theme, the patients described their relationship with the nurse as personal, yet not intrusive. The patients highlighted the importance of feeling trust in the relationship with the nurse, where they could talk about things they did not even share with their loved ones. On the other hand, if the patient didn’t feel trust towards the nurse, then continuity might be burdensome.

The patients described COPD as a shameful disease, even though some of them had never smoked. Knowing that the nurse has a duty of confidentiality made them feel trust and safety with the nurse. While stigmatisation was acknowledged, patients reported that nurses asked about their smoking history without conveying judgement or attributing blame for their COPD.

‘I never thought I would get COPD or asthma. But when I was sitting with my nurse and explained what I had worked with. I have been a smoker, of course. Yes. But I quit in 1985’. P10

Continuity in this personal relationship fostered a sense of trust and emotional safety, which was particularly important for patients who had not shared their diagnosis with family or friends. Some patients avoided such discussions to prevent causing worry, while others feared stigma. In this context, personal relationships with the nurse became a crucial source of support, allowing patients to speak openly in a confidential, non-judgmental environment and experience a respite where they could just be without fear of causing harm to their loved ones.

‘It’s more about how it is perceived in society, as it is still shameful. I don’t know if that’s the only reason I didn’t tell my loved ones. I think it’s also because they know what it was like with my husband so that the children and grandchildren don’t have to think about me in that way. There’s a lot behind it’ P13

The key to establishing a trusting relationship with a nurse might be small things, such as the patient being invited to sit next to the nurse rather than across the desk. Patients noted that this might be more challenging for the nurse, as they must turn their head, but this small gesture made the patients feel welcome. It could be compared to meeting with a friend in a coffee shop. Without this connection, trust with the nurse was hard to achieve for the patients.

‘Just being able to come to her room and sit next to her makes me feel safe, compared to when there was a desk between me and the nurse before’. P13

Continuity was perceived as beneficial by patients when it was founded on trust and a sense of safety. Establishing such trust requires a personal relationship with a nurse that patients regard as supportive of their healthcare experience. In the absence of trust, patients described continuity as burdensome, potentially leading to reluctance to seek care.

‘Before, I used to dread going. You just get scolded and told that you, you must do this and that and this and that, but I don’t get that now. Yes, there was a COPD nurse there too, but she should never have had that job. When you came, you got the finger wagging and you should breathe so much better, you have to stop smoking and you should do this and you should do that and… now listen to me, and that you will die prematurely’. P1

A personal relationship built on trust with a nurse was described by the patients as crucial in supporting long-term behavioural changes, such as smoking cessation. Patients described that having someone who genuinely cared about them and was invested in their well-being made it easier to stay motivated and committed to change. In contrast, when there was no trust in the relationship, patients found it difficult to fully engage in their care, especially when feelings of guilt or self-blame related to their condition were present.

‘It is what it is. I brought this upon myself; it’s because of my smoking. I have tried quitting smoking earlier without result 15 years ago but then there was no COPD nurse that I could talk to’. P4

## Discussion

The present study aimed to explore how patients with COPD experience COC in their encounters with a specialised asthma/COPD nurse in primary care. The findings demonstrate that patients attach significant value to COC. The patients described the value of not having to repeat themselves since the nurse already has a picture of them from previous encounters. Such a personal, yet professional relationship was described as central for fostering trust, which is especially vital considering that COPD is often perceived as a stigmatising condition, not always discussed with family or friends. Thus, contact with a dedicated nurse served as a unique point of respite for many patients, enabling them to discuss their condition openly in a non-judgmental environment. The three themes—*Meaningful care when encountering a dedicated nurse, Being acknowledged and listened to* and *Feeling trust in the relationship*—that emerged from the results are discussed below.

The first theme, *Meaningful care when encountering a dedicated nurse*, describes the importance of ongoing contact with an engaged nurse who coordinates care and serves as a stable point of reference. The experiences shared by patients emphasise the importance of having a stable point of contact and knowing who to reach out to when needed. This also resulted in patients not having to repeat themselves, since the nurse already had a clear picture of them from earlier encounters. This aligns with [36], who illustrated that patients value COC, especially the ability to consistently interact with the same nurse who manages and follows up on treatments. Similarly, Östman et al. [24] emphasised that patients appreciated meeting the same nurse at each visit, as it spared them from having to repeatedly explain their situation—something that otherwise made them feel uncared for. This is essential for ensuring long-term, responsible patient care by helping patients feel supported and by providing clear points of contact when needed. Such continuity may also contribute to more efficient use of healthcare resources.

However, some patients in our study described encounters with nurses who merely followed a checklist, lacked any personal connection, and were seen as shallow. Despite having a designated nurse, the absence of relational continuity led to care that felt impersonal and meaningless. This mirrors the findings of Östman et al. [24], in which patients felt like just another case, with nurses going through routines without addressing individual needs. Conversely, Ljungholm et al. [37] found that patients did not necessarily value a specific nurse but instead appreciated the nurse′s expertise.

The second theme, *Being acknowledged and listened to*, highlights the importance patients attach to feeling heard and recognised by nurses. A recurring topic was the value of the nurses’ relevant knowledge tailored to each patient’s needs. Patients emphasised that familiarity, established over repeated encounters and extended consultation times, facilitated a deeper understanding between them and the nurse, a dynamic they viewed as enhancing the perceived quality of care. Extended consultations were also associated with improved perceived quality of care [38], particularly among patients with chronic conditions [39]. Patients in our study also recalled previous encounters where nurses lacked sufficient knowledge or failed to provide essential information, such as proper inhaler techniques, although this appears to be inconsistently recognised in practice, according to Borge et al. [40].

To enhance tailored educational information suited to each patient, a partnership between a patient with COPD and an RN needs to be established [27, 41]. This was also found in our study, but this was not always the case in every encounter. According to Lundell et al. [28], barriers such as limited nurse knowledge, insufficient routines, and a lack of resources continue to impede effective patient education, potentially resulting in suboptimal outcomes. Lindh et al. [14] also emphasise the importance of enhancing education efforts for patients diagnosed with COPD.

In the third theme, *Feeling trust in the relationship*, trust emerged as a crucial element for patients, fostered by COC and by the presence of an RN who knew them well. Continuity enabled the growth of trust, providing a confidential space where patients could express vulnerabilities that often remained hidden from their loved ones. The duty of confidentiality by the nurses also helped patients talk about their previous smoking habits without judgement, allowing them to feel safe. This aligns with Lundell et al. (2020), who emphasised how trust helps patients overcome shame and stigma, especially when they haven’t shared their condition with others. Trust sets the foundation for a caring encounter, requiring both parties to meet as equals. As Halldórsdóttir and Hamrin [12] emphasised, trust bridges the nurse–patient relationship and is essential for genuine care. Holopainen et al. [42] further argue that such encounters can only emerge between individuals who engage in mutuality, allowing both to be fully themselves. To foster trust, nurses must recognise and understand the person behind the patient.

Despite its significance, trust has received limited attention according to Madawala et al. [43], underscoring the need for further research and development. Encounters with different nurses can hinder the building of trust, as highlighted in several studies. Gustafsson and Nordeman [44] found that while nurses aimed to build strong relationships, they often struggled. In contrast, a previous study by our research group, Roos et al. [25], reported that asthma/COPD RN often succeed in fostering a trusting relationship. However, not all encounters are caring. Without a caring encounter, patients may feel dismissed or unheard. A consistent, trusted nurse allows patients to speak freely without fear of judgement. In this context, a trusted nurse whom the patient meets consistently can play a crucial role in patient care, while an uncaring encounter may hinder it.

### Conclusion and implications for clinical practice

This study highlights that for patients with COPD, COC with a specialised asthma/COPD RN in primary care was both meaningful and supportive. Patients described that meeting with the same asthma/COPD RN over time fostered a sense of trust and security, allowing them to openly discuss sensitive issues, such as smoking, without fear of judgement. This ongoing relationship provides a safe and non-stigmatising environment. However, the findings also indicate that continuity alone does not guarantee high-quality care. The value of continuity is fundamentally linked to the presence of a genuine, trusting relationship. Without this relational depth, continuity can feel burdensome rather than beneficial. These findings underscore the importance of ensuring that COPD clinics in primary care are staffed with specialised asthma/COPD nurses who have sufficient time and resources to build long-term relationships. Such staffing enables clinical practice grounded in relational continuity, equipping nurses to foster trust, communicate without stigma, and deliver individualised care.

## Strengths and limitations

One strength of this study is that four different primary care centres from four distinct regions, in both rural and urban areas, were included, which makes these findings more applicable. Another strength is that patients of different backgrounds were interviewed, both regarding age but also years coping with COPD. These differences in experience were valuable for the study. The first author (SR) is a PhD student with limited prior training, while the co-authors are researchers experienced in qualitative design. The authors CMJ and JM also teach qualitative research methods at the university, both at advanced and doctoral levels. SR and MS continue to work part-time in primary care, which gives them valuable insight into the patients and the care they receive. To minimise the influence of the interviewer’s pre-understanding, a semi-structured interview guide with pre-arranged questions was used. The interview guide did not need any improvements after the first interview. After data collection, all interviews were discussed within the research group to ensure that participants had the opportunity to speak freely about their experiences. These discussions also helped identify whether any important topics were missing or needed further exploration during the interviews. Ongoing reflection during the process included the researchers′ understanding as the themes developed. The field notebook was also a helpful tool for the first author (SR) during interviews and data collection for reflecting on questions, thoughts and ideas.

Even though COC is an international phenomenon, this study was limited to a Swedish care context, which might affect the transferability of its findings to other clinical contexts. Therefore, readers should consider this if these findings are to be applied to other environments. Since the sampling was entrusted to COPD nurses, it could introduce bias, as they might have invited participants they perceived as cooperative or likely to provide beneficial responses, potentially altering the study’s results. However, the patients with COPD had varied experiences and perspectives, and not all were positive. Another limitation was that all interviews were conducted by telephone, which may have affected the depth and quality of the data. However, telephone interviews can make the conversation more anonymous and may, therefore, prevent the power imbalance between the interviewer and the interviewee [45]. This might also have been a strength, enabling participants to speak more freely because they could remain anonymous, as the interviewer had no prior contact with them.

## Data Availability

The qualitative data generated and/or analyzed in the current study are available upon reasonable requests from the corresponding author but are not publicly available because of privacy or ethical restrictions.
